# John D. Fisher, M. D.

**Published:** 1850-04

**Authors:** 


*John D. Fisher, M. D.*—Communicated for the Boston Medical and Surgical Journal.

Dr. Fisher died March 2d, aged 53. To the writer his death was sudden, and wholly unexpected. He had not heard of his illness; and it seems but a few days ago that he met him, and observing how much emaciated he was, and that he apparently was too feeble with safety to expose himself, even to the very mild winter day, asked him if he did not mean to run away from what remained of the season, and especially from our chilling spring atmosphere. My friend answered as he so generally did, that he was pretty well, and thought he might with entire safety remain where he was; and if he should be disappointed in his hope, he would go away for a time. Not long after, I learned from the newspapers that he was dead.

Dr. Fisher was emphatically a self-made man. That which created him —his own good spirit—remained true to its creation unto the end. He laid deeply the foundations of future eminence and usefulness in patient, laborious study of books, and careful record of what he saw, and of the thoughts or views to which direct observation gave birth. He went abroad and studied under the distinguished men of Europe that which had most occupied him at home; and those professional subjects which were novel, or with which his earlier pupilage and observation had made him but imperfectly acquainted. He was much interested in the study of smallpox, and gave to his profession a valuable and useful volume on that disease. Auscultation occupied much of his attention abroad, and he continued to deeply interested in its study and application, to the latest day of his professional life. He labored to extend auscultation to other diseases than those of the chest, having learned, by a large observation, how highly important are the physical signs of a disease to a correct diagnosis. Thus he employed auscultation in hydrocephalus, and sought to ascertain if any, and what were the precise cerebral sounds which accompany this disease. He published the results of his inquiries, and with a scientific precision and detail, which testified to the strength of his convictions concerning his statements. He early made trial of etherization in childbirth, and so satisfied was he of the entire safety and usefulness of this agent in that function, that he continued to employ it to his death. He most generally used chloroform. His habitual carefulness governed him in its employment. He always *dropped* it upon the ball of cotton which he used for its exhibition, that he might be sure of the quantity he employed, and frequently found ten or fifteen drops quite sufficient to produce such a degree of etherization as would make labor tolerable or painless.

It were easy to add to this enumeration of important offices to the sick, and valuable service to medicine, which were rendered to them by our deceased friend. There was one institution in which he was frequently found laboring. This was the Boston Society for Medical Improvement. He did not attend every meeting, for ill health, fatigue from professional toil, inclemency of weather, and other causes prevented this. But he rarely, if ever, came to our meetings, without bringing with him, either facts, or opinions, or both, which arrested and rewarded attention. He often read papers very carefully written, and of all necessary length, in which useful views were presented, or curious facts stated. These papers, and his larger contributions to medical literature, are now particularly remembered, as there is connected with them a fact, to which Dr. Fisher more than once referred, in conversation with the writer. This was the extreme difficulty or slowness with which he committed his thoughts to writing. "I have," he would say, "I have a perfectly distinct thought, or many thoughts ready for language, but the language comes so slowly, or seems so reluctant to come at all, that you cannot understand what a tedious business this writing is to me. If I could *paint* a thought, I should get on fast enough." In Dr. Fisher's family is a large development of the powers of drawing and of color, as is so well shown in the works of his gifted brother, the artist. Thought, says some one else, always brings with it its appropriate language; or, so to speak, always clothes itself in that dress which will best declare it to others. This may be true. But it is not at all difficult to understand, that what may be very well *said*, may require great labor to be well *written*.

Dr. Fisher, found time amid the interests of literary and practical life, to devote himself to objects which are sometimes considered to be rather of collateral than of immediate practical relation to medicine. And yet, strictly speaking, what is there relating to humanity which does not belong to it? He was one of the earliest advocates of the establishment of the Perkins Institution for the Blind. So early and so devotedly attached was he to this object, that he is considered to be its first suggester. For that which he was so instrumental in producing, he faithfully labored to his death. He was its Physician and its Vice President, and thus by perpetual personal agencies in its service—associations with its highest interests, it was his privilege and his happiness to mark and to minister to its steady progress, and to witness and rejoice in its entire success. During Dr. Howe's absence in Europe, the entire care of the institution devolved on Dr. Fisher. His whole service was thus faithfully devoted to its highest interest, and for it Dr. Fisher received no pecuniary compensation. It was a free-will offering of duty, and had its reward in itself.

Dr. Fisher has recently been elected one of the acting physicians of the Massachusetts General Hospital. How strikingly are both the proof and the illustration of the high consideration in which he was held by his profession, and the public, set forth in this important and distinguished appointment.

In this brief sketch of the prosessional life, and of some of its products, of a friend, it is not easy to forget the personal relations which that friend established, and which were alike grateful to him and those who enjoyed them. The moral nature declares itself in the familiar intercourse of life. Its fair development—that, namely, in which there is neither extravagant eccentricity, nor offensive radicalism—in which equanimity is the rule of every-day life, and courtesy and kindness its attendants—gives an interest to character and to conduct, which all men cheerfully recognize. The memory of an acquaintance of many years' standing does not bring to the mind of the writer one instance in which these elements of character were wanting in Dr. Fisher. He was happy in communicating to you what he knew, and of directly aiding you as a professional friend, whenever his special knowledge might serve you, or your patient. He would ask your counsel in his own cases; and then with entire confidence in you, would desire you to see them without special appointment with him, in order that you might observe effects of remedies, or other occurrences which had to you any particular interest. His heart, as well as his mind, was in his profession; and his patients and his brethren felt how useful and how grateful were their united agencies.

Dr. Fisher was quite remarkable for the simplicity and perfect repose which characterized his demeanor. He was justly sensible of what he thought wrong; but the expression of his feelings was not such as could tend to render the wrong greater. His position was too respectable to be hurt by what was untrue or unjust; and, so to speak, he could afford to command his temper. Who that knew him does not feel sad at his death? and yet did not death come in kindness to him, to draw to a calm close, a life devoted to duty, and to bring to everlasting rest a frame wasted by long disease, and so often exhausted by suffering?

The following notices of the last illness, and post-mortuary appearances in the case of Dr. Fisher, have been very kindly presented to the writer, by his friends, Drs. J. Bigelow, and J. B. S. Jackson.

It appears that Dr. Fisher visited Milton on Friday, 22d. Feb., and passed the night with Dr. Ware, of that place. On Saturday he had febrile symptoms and sore throat, but visited his patients, and in the evening attended the meeting of the Suffolk District Medical Society, feeling much indisposed at the time. On Sunday and Monday his symptoms continued, with increased debility and some vomiting. Dr. Whitney, of Dedham, who called on him on Monday, reports his aspect at that time to have been extremely morbid, and his throat much inflamed. On Tuesday, finding himself more ill, he sent for Dr. Bigelow, and inquired if he was likely to be more sick, since, in that case, he would wish to be removed to his brother's house, in Temple place. At this time his whole fauces, including both tonsils, velum and uvula, were of a dull red, turgid and glossy appearance, with a few specks of effused lymph on the tonsils. He had headache, difficult deglutition, a small pulse about 100, and much debility. He had taken a cathartic with moderate effect. Nitrate of silver was now applied to the parts of the throat most affected, and he was removed to his brother's house. On Wednesday morning he expressed himself better, especially in regard to deglutition. In the evening, feeling an increase of headache, he took, of his own accord, four grains of calomel, with eight of extract of colocynth, which operated moderately in the night, and on Thursday a diarrhœa of eight or ten discharges followed, which was not controlled under opiates and other remedies, and during which his strength sank rapidly. On Friday he appeared failing, pulse 130 to 140, small and feeble. During this time and until his death, there was no dyspnœa, slight cough, voice hoarse and abrupt, with difficult articulation, considerable dysphagia. He was visited by Drs. Jackson, sen. and jun. On Saturday there was slight aberration of mind, irregular action of heart, and failing pulse, and he died about midnight.

On dissection, there was found an acute inflammation of the larynx. The glottis upon the left side was considerably tumefied, though quite soft, and scarcely reddened; cellular tissue about the muscles, to some extent, looked as if infiltrated with pus, though very little could be forced out; œdematous swelling about the ventricle upon this side, so that its cavity was nearly closed. Right side similarly, though much less affected. Disease limited to upper half of larynx, the passage being apparently sufficiently free for the admission of air. Mucous membrane of pharynx of a dusky-red color; but nowhere any effusion of lymph.

At the apex of the right lung, posteriorly, there was an old pleural adhesion; the surface of the organ was much puckered; and beneath it were several white, opaque, dryish, firm masses, partly cretaceous, and about the size of peas. At the corresponding part of the left lung, there was also an old adhesion; the surface of the lung had a wilted appearance, and beneath it was found a grayish granulated deposit, not larger than a pea. And these were all the remains that were to be found of tubercular disease; the lungs being otherwise sufficiently healthy, as were the bronchial glands.

W. CHANNING.

